# Estimating Polar Bear (*Ursus maritimus*) Age Based on an Epigenetic DNA Methylation Clock

**DOI:** 10.1002/ece3.71870

**Published:** 2025-07-28

**Authors:** Susannah P. Woodruff, Milda Milčiūtė, Juozas Gordevičius, Robert Brooke, Todd C. Atwood

**Affiliations:** ^1^ Marine Mammals Management US Fish and Wildlife Service Anchorage Alaska USA; ^2^ Epigenetic Development Clock Foundation Torrance California USA; ^3^ U.S. Geological Survey Alaska Science Center Anchorage Alaska USA

**Keywords:** age estimation, chronological age, DNA methylation, epigenetics, polar bear, wildlife management

## Abstract

Knowledge of animal age is essential to wildlife managers for obtaining meaningful and accurate insights into demographic parameters. A common approach to aging wildlife, including bears (*Ursus* spp.), has been extracting a tooth during physical capture and counting the cementum annuli. Limitations to tooth‐based aging include questionable accuracy and differing results based on the observer and laboratory. DNA methylation‐based epigenetic aging clocks have been developed for many species but not yet for polar bears ( *Ursus maritimus* ). We generated DNA methylation data from whole blood samples (*n* = 109) obtained during live capture operations from polar bears of known age in the Chukchi Sea and southern Beaufort Sea subpopulations. We used these samples to calibrate a species‐specific epigenetic clock to estimate polar bear chronological age from DNA methylation (DNAm) age. The final polar bear clock was highly accurate (*r* = 0.97) with a median absolute error of approximately 9 months. We applied the polar bear clock to 74 blood samples from live‐captured polar bears with a cementum annuli‐estimated age. Predicted age estimates for these bears ranged from 1.43 to 18.63 years compared to the estimated tooth age range of 3.23–25.27. These epigenetic clocks can be used for polar bear research and management where accurate estimates of age are needed for estimating demographic parameters.

## Graphical abstract

We developed a DNA methylation‐based epigenetic aging clock for polar bears ( *Ursus maritimus* ) using whole blood samples from polar bears of known age. The final polar bear clock was highly accurate (*r* = 0.97) with a median absolute error of approximately 9 months.

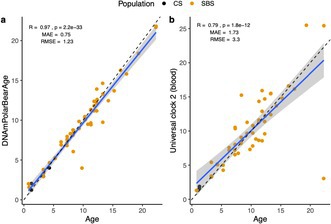

## 1 Introduction

Understanding population demographic metrics, such as abundance, survival, and recruitment, is central to wildlife management (Buckland and Johnston 2017; Wikenros et al. 2021). These metrics are, however, often costly and difficult to obtain, particularly for low‐density, wide‐ranging species, which are inherently difficult to monitor due to low detection rates (Morin et al. 2022; Woodruff et al. 2016). Because vital rates often vary by age, known age is necessary for projection models to estimate population growth rates (Eberhardt 1985; Paterson et al. 2022). Further, knowing the age structure of a population can be invaluable for predicting future population performance due to effects of age‐specific survival or reproduction (Arso Civil et al. 2019; Bouwhuis et al. 2012). Without knowing the age of sampled individuals, inferences are inherently limited, requiring assumptions that may be misleading.

Traditional age estimation methods include the use of phenotypic traits (e.g., body or antler size [e.g., Gee et al. 2014]); indices based on tooth eruption, staining, and wear patterns (e.g., Zhang et al. 2024); or counting cementum annuli of teeth extracted from captured or harvested animals (e.g., Christensen‐Dalsgaard et al. 2010; Costello et al. 2004). Tooth extraction has been used extensively in wildlife research and monitoring despite the questionable accuracy of the method and the debate surrounding the ethics of tooth removal on live animals (Festa‐Bianchet et al. 2002; Nelson 2001). Other limitations to tooth‐based aging include small sample sizes, the lack of a representative sample, and the expense of physical capture. Additionally, across species, the accuracy of cementum annuli‐determined ages varies based on factors including species and investigator experience and generally decreases as animals age (e.g., Costello et al. 2004; Hamlin et al. 2000; Harshyne et al. 1998; Veiberg et al. 2020).

Aging via analysis of the level of DNA methylation (DNAm age) is an emerging method gaining acceptance as a less‐invasive and more accurate method than traditional methods (Le Clercq et al. 2023; Newediuk et al. 2025). DNA methylation is the addition of methyl groups (CH_3_) to specific cytosine‐guanine (CpG) sites resulting in epigenetic changes to the genome. The methylation patterns found at these CpG sites can be used to develop an “epigenetic clock” (Horvath and Raj 2018). DNA methylation levels are highly correlated with chronological age (i.e., time since birth; Christiansen et al. 2016; Horvath and Raj 2018), and a clock trained with samples from known‐age individuals can be used to predict the age of individuals of unknown age (Barratclough et al. 2024; Bors et al. 2021). Methylation levels are also correlated with biological age (i.e., the age cells appear to be; Chen et al. 2016), and the difference between chronological and biological age is indicative of health and life expectancy (Chen et al. 2016; Dugué et al. 2018; Marioni et al. 2018). An individual's level of DNA methylation is influenced by factors including nutrition, stress, age, and the environment (Ryan et al. 2020).

DNAm age, or epigenetic, clocks have provided accurate ages in > 125 non‐human mammalian species (e.g., Arneson et al. 2022; Czajka et al. 2024; Nakamura et al. 2023). This ultimately led to development of a universal mammalian clock (Arneson et al. 2022) and several multi‐species clocks (Caulton et al. 2022; Robeck, Fei, Lu, et al. 2021; Robeck et al. 2023). Universal and multi‐species clocks are valuable, yet species‐specific, or even population or clade‐specific (Nakamura et al. 2023), clocks are more accurate (Field et al. 2018; Peters et al. 2023; Zhang et al. 2019).

Polar bears ( *Ursus maritimus* ) occur in 20 subpopulations throughout the seasonally and permanently ice‐covered marine waters and adjacent coastal areas of the Arctic. They are a sea ice dependent species (Amstrup et al. 2008; Wilson et al. 2014, 2016) listed as threatened under the United States (US) Endangered Species Act since 2008 (ESA; 73 FR 28212), depleted under the US Marine Mammal Protection Act (MMPA; 16 U.S.C. §§ 1361 *et seq*.), a species of Special Concern by the Committee on the Status of Endangered Wildlife in Canada (COSEWIC 2018), and Vulnerable by the International Union for the Conservation of Nature (Regehr et al. 2018). Loss of sea ice habitat as a result of climate warming is the primary threat to polar bears (73 FR 28212). Polar bears also have significant cultural importance and are an important subsistence species harvested by many Indigenous peoples throughout the Arctic (Born et al. 2011; Schliebe et al. 2006; Voorhees et al. 2014). Monitoring population demographic status is critical, particularly for harvested, at‐risk species (Laidre et al. 2015; Regehr et al. 2018) and is also mandated by both the ESA (i.e., 5‐year review; 16 USC 1531 *et seq*.) and MMPA (i.e., stock assessment report; 16 U.S.C. §§ 1361 *et seq*.).

The US (Alaska) shares two subpopulations, both managed by the US Fish and Wildlife Service (FWS): Chukchi Sea (CS), which is shared with Russia, and southern Beaufort Sea (SBS), which is shared with Canada. These subpopulations share a geographical boundary delineated at Icy Cape, Alaska, and have some overlap in distribution (Amstrup et al. 2000, 2004; Garner et al. 1990; Scharf et al. 2019). Both subpopulations have long‐term capture datasets dating back to the 1980s. Extracting a tooth during physical capture from newly captured independent bears (i.e., not with their mother) for cementum annuli aging is common practice. Results of studies assessing the accuracy of this method from the United States, Canada, and Norway have indicated relatively low accuracy with 32%–45% (Hensel and Sorensen 1980), 58%–75% (Calvert and Ramsay 1998), and 50%–53% (Christensen‐Dalsgaard et al. 2010), respectively, aged correctly to within 1 year. Such issues highlight the need to develop better methods of aging.

Here we present a species‐specific DNAm age estimation model for polar bears based on methylation levels of blood‐derived DNA. The development of a species‐specific epigenetic aging clock requires samples from known‐age individuals for calibration. Leveraging long‐term datasets, we measured methylation levels in blood samples collected during the capture of known‐age wild polar bears from the CS and SBS subpopulations to build the DNAm age predictive model. We then used the model to estimate the age of polar bears with age previously estimated by cementum‐annuli methods (i.e., tooth‐aged). Our epigenetic clock complements epigenetic clocks previously developed for polar bears in other subpopulations (Newediuk et al. 2024, 2025) and will aid polar bear conservation and management through informing research, such as age‐specific vital rates used in population models and age‐ordering to facilitate kinship and pedigree analysis.

## 2 Materials and Methods

### 2.1 Sample Collection

Blood samples were collected from wild polar bears in the CS (2008–2017) and SBS (1989–2016) subpopulations as part of capture and release programs (e.g., Rode et al. 2014). Blood samples were collected typically via the jugular vein or femoral vein into vacuum tubes containing the anticoagulant EDTA. Blood (1–1.5 mL) was frozen as whole blood in 2 mL cryovials and transferred to a −80°C freezer upon returning from the field. A premolar tooth was also extracted from independent bears (i.e., not with their mother) upon initial capture for cementum annuli age estimation. Teeth were stored in a coin envelope or Whirlpak at room temperature. All procedures involving animal handling were conducted following guidelines approved by the Institutional Animal Care and Use Committees for US Geological Survey (USGS) in the SBS and FWS in the CS.

Because these were wild bears and actual birth dates are unknown (Derocher et al. 1992; Van de Velde et al. 2003), all bears were given a birth date of 1 January. For methylation analysis, we assembled two datasets from an initial total of 192 blood samples (96 each from CS and SBS; Table 1, Table S1). For our first dataset for DNAm age calibration (hereafter, calibration samples; *n* = 89), we used samples from polar bears of known chronological age determined by initial capture as dependent cub‐of‐the‐year (age zero), yearling, or 2‐year‐old, and subsequent recaptures of these individuals.

**TABLE 1:** Number and description of polar bear whole blood samples collected from two subpopulations, southern Beaufort Sea (SBS) and Chukchi Sea (CS) during 1989–2017. Samples used for epigenetic clock calibration were from bears with a known age (0.75–23.32 years), that is those initially captured as either a dependent cub‐of‐the year (age zero), yearling, or 2‐year old. Test samples were from bears with an estimated age (i.e., from cementum annuli; 3.23–25.27 years).

	Calibration samples (known age)		Test samples (tooth age)	
	SBS	CS	SBS	CS
Initial	93	20	3	76
Dropped	4	0	0	5
Final	89	20	3	71
Male	41 (24)	8 (6)	0	37 (27)
Female	48 (29)	12 (7)	3 (1)	34 (27)

*Note:* Initial = original number or samples submitted for analysis; dropped = number of samples dropped due to failure to amplify or outliers; final = final number of samples for each dataset (i.e., calibration or test); male and female represent the final number of samples from each sex. Because multiple samples came from the same individual, the number of individuals is in parentheses.

To test the performance of the model, we used 74 whole blood samples (hereafter, test samples) from bears with ages estimated via tooth cementum annuli analysis (SBS: *n* = 3; CS: *n* = 71; Table 1, Table S1). Cementum annuli aging was conducted by multiple laboratories (Matson's, Manhattan, Montana, USA; Alaska Department of Fish and Game, Fairbanks, Alaska, USA; Canadian Wildlife Service, Edmonton, Alberta, Canada) but generally followed the same procedures (see Christensen‐Dalsgaard et al. 2010). Each tooth was assessed independently by two people, and we included the sample only if the age estimate was the same between the two people. For bears subsequently recaptured, we derived the age from the tooth‐estimated age from the first capture. We selected samples using estimated tooth age in an attempt to have an even age distribution for our test samples and for comparison with the DNAm predicted age, although we recognize that, as mentioned above, tooth age accuracy is relatively low (Christensen‐Dalsgaard et al. 2010). We also used these samples to proof the model, that is, were samples collected in later years predicted to be older by the epigenetic clock? We used longitudinal samples (i.e., the same individual sampled in multiple years) in both sample sets to help validate expected changes as individuals age. Both datasets reflect typical polar bear age‐at‐capture distribution (Derocher 2005; Henriksen et al. 1997; Peacock et al. 2013) and expected polar bear age structure.

### 2.2 DNA Extraction and Methylation Analysis

We extracted DNA from the whole blood samples using a HMW DNA extraction kit (Zymo Research, Irvine, CA, USA) according to the manufacturer's protocols. Following extraction, genomic DNA was subjected to bisulfite conversion using the EZ DNA Methylation Kit (Zymo Research, Irvine, CA, USA), which is standard for downstream processing with the HorvathMammalMethylChip40 array. Bisulfite treatment converts unmethylated cytosines to uracil while leaving methylated cytosines unchanged, allowing for the accurate detection of methylation status at individual CpG sites. Concentrations of DNA extract ranged from 20 to 50 ng/uL per individual and were loaded into a 96‐well plate for analysis.

We generated DNA methylation data using a custom Infinium array (Illumina, San Diego, CA, USA) assembled with 37,492 CpG probes (HorvathMammalMethylChip40; Arneson et al. 2022). Raw data were normalized using the *SeSAMe* package (Zhou et al. 2018), resulting in a methylation estimate (beta value) corresponding to each array probe for every individual in the dataset and a detection *p*‐value corresponding to the confidence in the normalized beta value. Beta values are derived from the ratio of the fluorescence intensity of a methylated probe for a specific CpG to the total overall probe intensity (the sum of the signal from both the methylated and unmethylated probes plus a constant; Du et al. 2010). Beta values range from zero to one, with a value of zero indicating that no copies of the gene were methylated (Zhou et al. 2018). We conducted all analyses in R v.4.2.0. (R core team 2022).

We performed a series of quality control steps to identify outliers. Probes with both methylated and unmethylated channels reporting background signal levels with the *p*‐value threshold of *p* > 0.01 were marked as failed. Small *p*‐values indicate good quality probes. A high detection *p*‐value (*p* > 0.01) suggests the signal from the probe is not significantly different from the background noise, indicating a likely failure of that specific probe. We removed these probes early to ensure the integrity of our dataset. Additionally, we counted the proportion of failed probes for each sample. Samples with more than 30% of failed probes were considered outliers. Principal component analysis (PCA) is a data dimensionality reduction method that helps identify the main axes of variance within the dataset (Brems 2022). We computed PCA of the centered, unnormalized beta‐value matrix. Additionally, we calculated unsupervised hierarchical clustering using inter‐array correlation of the samples (Pai 2021). We expect pairwise correlations of > 0.95 from samples of the same tissue type under the same conditions; values < 0.8 indicate outliers or technical issues. Samples greater than two standard deviations of the first three principal components, or more than two standard deviations from the mean for PCA and hierarchical clustering, respectively, were marked as outliers.

### 2.3 Clock Development

Initially we applied universal clocks to our samples (*n* = 91 after removing outliers). Universal clocks may be non‐species and non‐tissue specific (i.e., Universal Pan Tissue Clock) or tissue specific but applicable to > 185 mammalian species (e.g., Universal Blood Clock 2; Lu et al. 2023). Although these clocks performed reasonably well with our samples (see (3) results), we attempted to improve the precision by developing a polar bear specific clock.

We constructed an elastic net clock model with 109 known age samples using the R package glmnet (Friedman et al. 2010). We used a log‐linear age transformation that was dependent on a mean age at sexual maturity of 4.75 years for both polar bear sexes and a gestation time of 0.18 years (AnAge 2023) as follows. Let *Yik* denote the transformed age of a sample *i* from a polar bear population *k*, mean age at sexual maturity for polar bears = *a*_*k*_ and the gestation time = *g*_*k*_. We calculated the log‐linear transformation for polar bear age, *Y*_*ik*_, as shown in (Equation 1):

(1) $$Y_{\mathrm{ik}}=\left\{\begin{array}{ll} \log\left(\frac{X_{\mathrm{ik}}+g_{k}}{1.5a_{k}+g_{k}}\right), & X_{\mathrm{ik}}\leq 1.5a_{k}\\ \frac{X_{\mathrm{ik}}-1.5a_{k}}{1.5a_{k}+g_{k}}, & X_{\mathrm{ik}}>1.5a_{k} \end{array}\right.$$

The transformation follows a logarithmic form when the chronological age is young and shifts to a linear form as age increases. This approach accounts for the faster epigenetic aging that occurs during developmental stages (Lu et al. 2023). Within the calibration dataset (i.e., known age bears), we randomly partitioned the individuals into training (70%) and validation (30%) sets using stratified sampling based on age. For individuals sampled multiple times, all samples from the same individual were restricted to the same set to avoid data overfitting. Specifically, we selected one sample per bear to define the partition and then assigned all corresponding samples from each individual to either the training or validation set based on this partitioning. Prior to training the model, beta values were filtered to include only those probes with a detection *p*‐value < 0.05 in 95% of the samples. We chose this cutoff value in order to retain probes that were reliably measured in the vast majority of our samples and maintain a larger pool of potentially informative probes for the model, while still ensuring these probes were generally well‐measured across the model. Additionally, principal component‐based preprocessing, along with matrix scaling and centering, was applied to beta values to address highly correlating CpGs and mitigate the batch effects arising from different subpopulations (Higgins‐Chen et al. 2022). The training dataset was used to train the clock using five times repeated seven‐fold cross validation. In each partition, we selected the best predictor using the root mean squared error (RMSE) metric. To evaluate the effectiveness of the clock, we assessed Pearson's correlation coefficient (*r*) and median absolute error (MAE) between known age and DNAm predicted age. The final polar epigenetic clock was computed using 65 principal components (Tables S2 and S3). The final elastic net regression model was chosen by the lowest RMSE value and had an alpha of 0.9 and a lambda of 0. We applied the final model to the internal validation dataset and independent test samples (i.e., tooth age bears; *n* = 74) to estimate DNAm ages. Additionally, because the time our samples were in storage prior to our analyses ranged from 6 to 36 years, we used linear regression to test whether there was a correlation between the age of the sample (i.e., date of collection) and the residuals (i.e., the difference between known and predicted age).

## 3 Results

### 3.1 The Dataset

**FIGURE 1:**
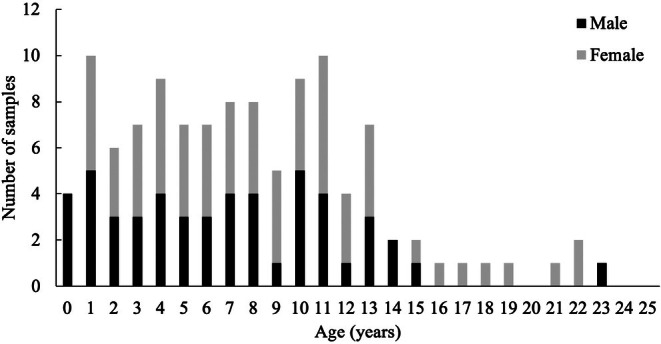
Sex and age distribution of samples from polar bears captured in the Chukchi Sea (2008–2017; *n* = 20) and southern Beaufort Sea (1989–2016; *n* = 93) subpopulations by sex for calibration samples (i.e., known age).

High quality DNA methylation profiles were generated from 192 wild polar bear blood samples from the two subpopulations. Seven technical outliers were identified through hierarchical clustering and removed from the analysis. The final calibration dataset included 109 blood samples consisting of 89 samples from SBS and 20 from CS, with known ages of animals ranging from 0.75 to 23.32 years (mean ± SD = 8.18 ± 5.23 Figure 1; Table 1, Table S1). Samples were from 69 unique individuals; 62 were samples from the same individuals sampled over multiple years. Thirteen individuals were sampled in 2 years and three individuals in each of three, four, and five different years.

The final dataset of test (i.e., tooth‐aged) samples included 74 samples from 54 unique individuals; 34 were samples from the same individuals sampled over multiple years. Twelve, two, and one individuals were sampled in two, three, and four different years, respectively. Samples from the 15 tooth‐aged individuals with multiple captures were sampled over time periods ranging from 1 to 9 years. The average difference between time elapsed and DNAm age estimates for test samples was 1.75 years (SD = 1.65; range = −1.89–4.66 years; Figure S1). Age generally increased in successive time points, although for three individuals, age increased less than expected (i.e., less than the interval between capture events) or decreased.

### 3.2 The Epigenetic Aging Clock

**FIGURE 2:**
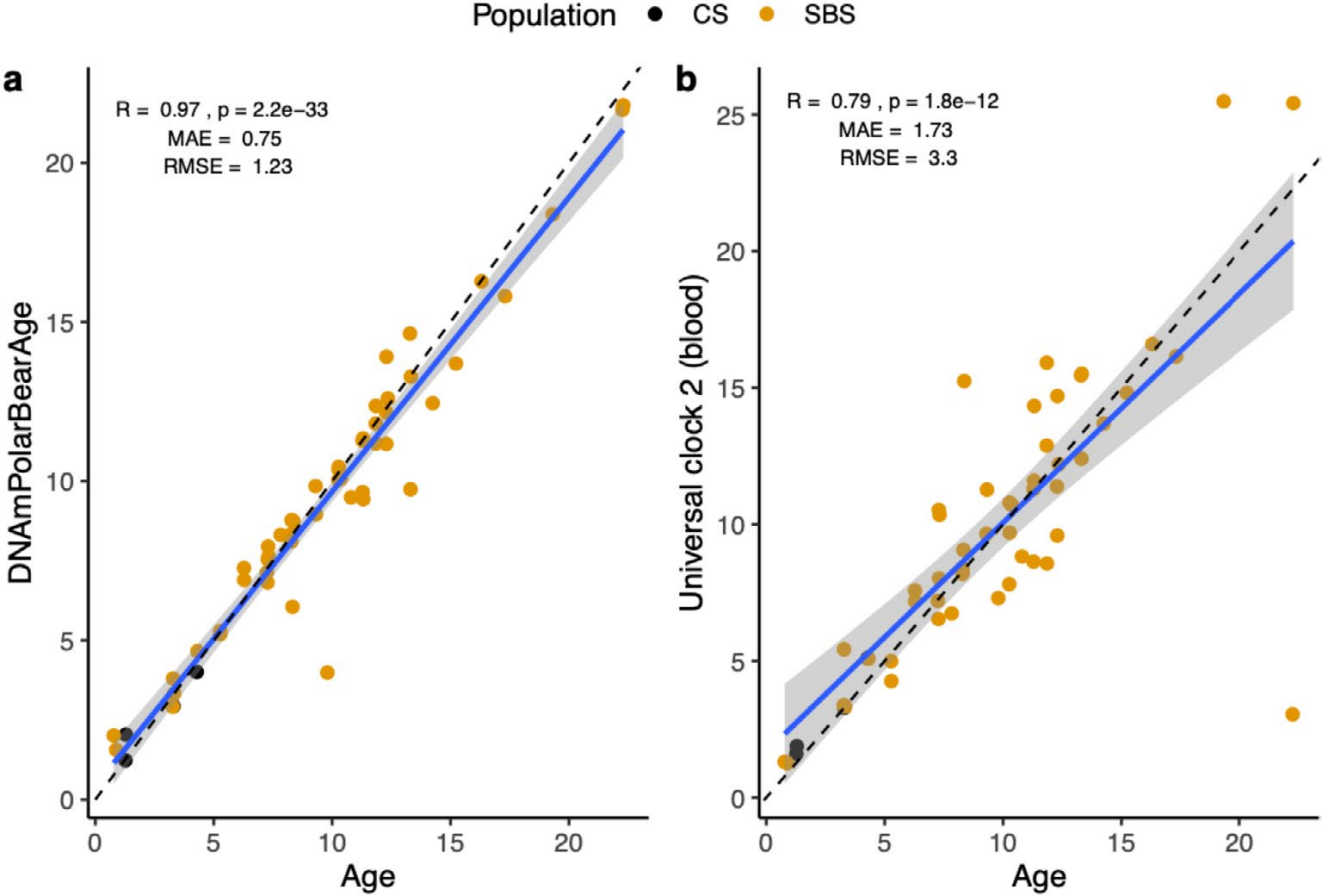
Scatter plots of predicted age (years; DNAmPolarBearAge) and known chronological age in validation dataset for (a) the polar bear age predictor and (b) the universal clock 2 blood predictor. The solid blue line represents the linear regression line fit to the data points, and the gray shaded area indicates the 95% confidence interval around this regression line. The dashed line represents a 1:1 reference line where predicted age = actual age; the median absolute error (MAE) is visually represented by the distance between the solid and dashed lines.

The species‐specific polar bear clock demonstrated high accuracy across the cross‐validation subset (i.e., 5 × 7‐fold from 70% of 109 samples) with *r* = 0.80, MAE = 1.99, and RMSE = 3.21. For the validation dataset, that is, the known age samples that were not used in the training of the clock (*n* = 33), *r* = 0.97, MAE = 0.75, RMSE = 1.23 (Figure 2a), compared to *r* = 0.79, MAE = 1.73, and RMSE = 3.3 for the Universal Blood Clock (Figure 2b). Post hoc, we used the validation dataset to examine the correlation between the predicted ages generated by the polar bear clock and those from four other universal clocks and found a strong correlation (*r* = 0.77–0.79, Figure S2).

**FIGURE 3:**
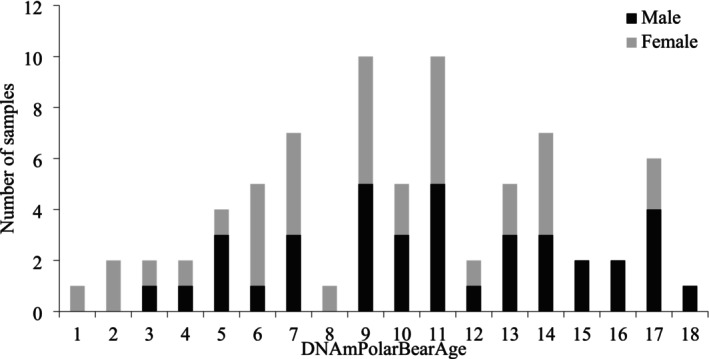
The predicted age (DNAmPolarBearAge) for test samples collected from polar bears during live capture in the Chukchi Sea from 2008 to 2017 (*n* = 71; 54 individuals) and southern Beaufort Sea from 2004 to 2007 (*n* = 3; 1 individual).

**FIGURE 4:**
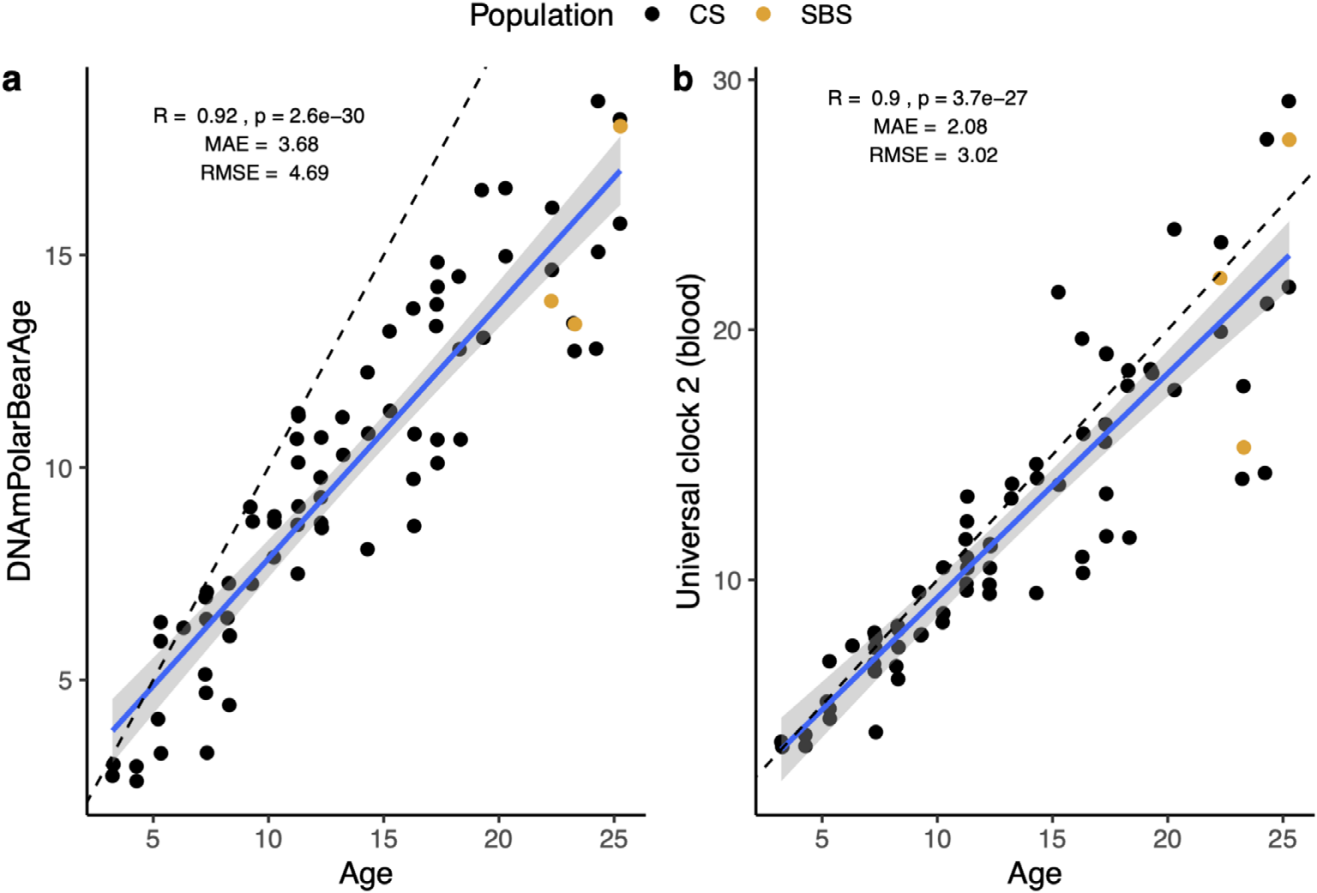
Predicted age (DNAmPolarBearAge) versus tooth age for (a) the polar bear specific clock and (b) the universal blood clock for blood samples collected from polar bears during live capture in the Chukchi Sea (2008–2017; *n* = 71; 54 individuals) and southern Beaufort Sea subpopulations (2004–2007; *n* = 3; 1 individual). The solid blue line represents the linear regression line fit to the data points, and the gray shaded area indicates the 95% confidence interval around this regression line. The dashed line represents a 1:1 reference line where predicted age = actual age. The median absolute error (MAE) is visually represented by the distance between the solid and dashed lines.

Predicted ages from the test samples (i.e., samples from tooth‐aged bears) ranged from 1.43 to 18.63 ± 0.75 years (Figure 3) compared to the estimated tooth age range of 3.23–25.27. The comparison of tooth age versus DNAm age showed a higher correlation (*r* = 0.92) but a higher error (MAE = 3.68 years; Figure 4a) compared to Universal Blood Clock 2 (*r* = 0.90 and MAE = 2.1; Figure 4b). The difference between DNAm age and estimated tooth age was larger for older individuals (as predicted by tooth age).

Results of the linear regression analysis indicated there was no correlation between the age of the sample (i.e., date of collection) and the residuals (i.e., the difference between known and predicted age) (Beta = −0.018, *p*‐value = 0.71). Similarly, a two‐sided *t*‐test comparing residuals between subpopulations showed no significant difference (*p*‐value = 0.46), indicating that prediction errors were not biased toward one subpopulation or processing batch.

## 4 Discussion

We developed a robust epigenetic clock to estimate age from two wild polar bear subpopulations using blood samples. There was a correlation between epigenetic (DNAm) and chronological age throughout the polar bear lifespan. Polar bears in the wild have a lifespan of approximately 25 years (Rode and Stirling 2018), thus the clock estimates age to ±3% of the polar bear lifespan, although we caution that age estimates may be less accurate for older individuals. Advantages to this method include obtaining more accurate age estimates compared to cementum annuli‐derived ages and leveraging samples likely already routinely collected (e.g., blood, tissue) during capture, as opposed to pulling teeth. Further, archived samples or previously extracted DNA may be used for DNAm analysis, extending the value of existing samples and saving money on DNA extraction costs. Our results complement other polar bear clocks (Newediuk et al. 2024, 2025) and provide additional support for the use of DNAm methods to estimate the age of wild mammals (De Paoli‐Iseppi et al. 2017).

Our DNAm method estimates polar bear age with a MAE of 0.75 years. In contrast, accuracy of cementum annuli for aging polar bears to within 1 year of actual age has ranged from 32% to 75% (Calvert and Ramsay 1998; Christensen‐Dalsgaard et al. 2010; Hensel and Sorensen 1980). Cementum annuli age estimates are less accurate when made by less experienced observers (Christensen‐Dalsgaard et al. 2010; Hensel and Sorensen 1980; McLaughlin et al. 1990) and can vary between laboratories by > 10 years (Christensen‐Dalsgaard et al. 2010). For example, in one lab, multiple female polar bears were aged at ~10 years, while a second laboratory aged them at > 20 years old (Christensen‐Dalsgaard et al. 2010). Our method removes these sources of error. We do, however, acknowledge a potential source of technical error, given that all but three of the tooth‐aged samples were run on one plate, and the majority of our known age samples were run on another plate. It is possible that some of the differences we observed between our internal validation samples (i.e., known age samples not used to calibrate the clock) and the tooth‐aged test samples could be due to technical variation between plates (see below for additional (4) discussion).

The cross validation showed a pattern of age overestimation for younger bears and underestimation for older bears, similar to patterns shown in other species (e.g., Bors et al. 2021; Peters et al. 2023; Polanowski et al. 2014). Also similar to others, errors increased with increasing age (e.g., Barratclough et al. 2024; Peters et al. 2023). This is likely due to the distribution of our calibration samples with fewer samples from individuals > 15 years old (Figure 1). Our calibration samples do, however, follow typical polar bear age‐at‐capture distribution (Derocher 2005; Henriksen et al. 1997; Peacock et al. 2013) and expected polar bear age structure, and thus fewer samples from older individuals are expected. We also acknowledge that non‐linear age‐related changes in DNA methylation are well documented (e.g., Alisch et al. 2012; Horvath and Raj 2018) and caution that age estimates may be less accurate for older individuals given our limited sample size (i.e., few older individuals) for our clock validation. Previous research indicates clock accuracy changes with age (e.g., Simpkin et al. 2016 [humans]; Newediuk et al. 2025 [polar bears]).

Development of a species‐specific epigenetic aging clock requires samples from known‐age individuals for calibration, which can be a limiting factor (Mayne et al. 2021) making the universal clock more appealing. However, whereas pan‐mammalian clocks have been shown to predict age with relative accuracy, our results provide additional support for species‐specific clocks, which have been shown to outperform universal clocks (e.g., this study, Peters et al. 2023; Czajka et al. 2024). Mayne et al. (2021) provided guidelines to determine the necessary sample size and age distribution to ensure the development of a useful species‐specific epigenetic clock. They suggest a minimum sample size of 70 (Mayne et al. 2021), although others suggest 40 samples for calibration is sufficient (Le Clercq et al. 2023). Ensuring relatively uniform age and sex distribution is also paramount to accurate clock development (Mayne et al. 2021). Initially, we attempted to develop an epigenetic clock with blood samples from tooth age (*n* = 76) and known‐age individuals (*n* = 20) all from CS bears. Correlation was strong in this universal clock (*r* = 0.91), but the MAE was higher than desired (3.54 years). We took advantage of archived samples from a long‐term USGS capture dataset and increased our sample size by adding samples from known‐age SBS bears, which substantially improved our predictor (*r* = 0.97; MAE = 0.75 years).

The addition of these samples also created some complications. First, 82% (*n* = 89) of our samples used to train the predictor came from a single subpopulation (SBS); however, the rate of methylation varies both within and between subpopulations due to varying environmental conditions (Lemaître et al. 2022; Nussey et al. 2007), although previous polar bear DNA methylation analysis indicated similar DNA methylation patterns across subpopulations (Newediuk et al. 2025). We initially hypothesized there may be differences in methylation levels between the two subpopulations given observed differences in survival and abundance, body mass and condition, and recruitment (Bromaghin et al. 2015, 2021; Regehr et al. 2010, 2018; Rode et al. 2010, 2014, 2021). Our results indicated CS bears were biologically younger than SBS bears as we expected; however, because we ran these samples in separate batches by subpopulation (i.e., all CS in the first batch, all SBS in the second batch), we were unable to distinguish any between‐subpopulation differences in biological age from a batch effect. This was a result of needing a larger sample size and capitalizing on the SBS dataset, which was run in a second batch. Had we initially known we were going to need a larger sample size and would use samples from another subpopulation, we would have randomly mixed the samples in the two batches. We implemented PCA‐based preprocessing to train the predictor and are confident our epigenetic clock is robust for estimating age in polar bears; however, we recommend randomizing samples from multiple subpopulations across processing batches to get better insights into subpopulation differences in the future. Second, we focused our clock validation on only whole blood, largely because we had a robust set of archived, longitudinal samples from known age bears. We do, however, expect there may be an increase in biopsy darting and noninvasive sampling (e.g., scat, saliva) in the future given the uncertainty of the feasibility of on‐ice physical capture due to declining sea ice (Wilson et al. 2019) and the continued opposition to capture (Von Duyke et al. 2017; Wong et al. 2017). When we compared the performance of our blood‐based polar bear clock to existing universal clocks, we included clocks developed for different tissue sources (e.g., Universal Skin Clock2: *r* = 0.71, MAE = 2.30 years; Universal Pan Tissue: *r* = 0.79, MAE = 4.9 years) to provide a broader context for its accuracy. As expected, our species‐specific blood clock generally outperformed these universal clocks, including those designed for other tissues, highlighting the importance of tissue‐specific and species‐specific clock development for optimal accuracy. Several studies indicated blood epigenetic clocks were more accurate compared to skin clocks in cetaceans and pinnipeds (e.g., Barratclough et al. 2021; Robeck, Fei, Haghani, et al. 2021). Analysis of DNA obtained from non‐invasively collected samples (e.g., scat) is complicated by the presence of non‐host DNA and the lower quantity of DNA in the sample (Chiou and Bergey 2018), although recent research with scat samples shows promise (Hanski et al. 2024; Yagi et al. 2024).

Our polar bear epigenetic clock provides wildlife managers and researchers with an accurate and humane aging method, eliminating the need for tooth extraction of live‐captured animals. Validation of this epigenetic clock with high quality DNA from blood samples facilitates the next phase of our project during which we are collecting and analyzing multiple sample types (e.g., blood, tissue, scat, saliva, hair) from each captured bear to validate DNAm clocks for other sample types in the CS and SBS subpopulations. As human‐induced climate change and other anthropogenic stressors continue to threaten polar bear persistence and the loss of sea ice compromises our ability to conduct traditional monitoring (i.e., capture and radiocollaring), developing methods that enable managers to continue collecting data (e.g., age distribution) to inform sustainable harvest and population trajectories is crucial. This clock may broadly apply to other polar bear subpopulations, though it would be judicious to test the clock with known age samples from different subpopulations to ensure any differences between subpopulations do not affect age estimates.

## Author Contributions

**Susannah P. Woodruff:** conceptualization (lead), funding acquisition (lead), methodology (supporting), resources (lead), writing – original draft (lead). **Todd C. Atwood:** resources (equal), writing – review and editing (equal). **Milda Milčiūtė:** formal analysis (equal), methodology (equal), validation (equal), writing – review and editing (equal). **Juozas Gordevičius:** formal analysis (equal), methodology (equal), writing – review and editing (equal). **Robert Brooke:** conceptualization (equal), methodology (equal), project administration (equal), supervision (equal), writing – review and editing (equal).

## Conflicts of Interest

The authors declare no conflicts of interest.

## Supporting information


**Appendix S1.** ece371870‐sup‐0001‐AppendixS1

**Table S2.** ece371870‐sup‐0002‐TableS2

**Table S3.** ece371870‐sup‐0003‐TableS3

## Data Availability

Normalized data are uploaded as Supporting Information. The mammalian methylation array is available from the non‐profit Epigenetic Clock Development Foundation.
