# Supplementary material for: Estimating Polar Bear (Ursus maritimus) Age Based on an Epigenetic DNA Methylation Clock
Source: Ecol Evol. 2025 Jul 28;15(8):e71870. doi: 10.1002/ece3.71870 (PMC12304446; doi:10.1002/ece3.71870)
Supplement: Supplementary file 1 — Appendix S1. ece371870‐sup‐0001‐AppendixS1 [file ECE3-15-e71870-s003.docx]

**Estimating polar bear (*Ursus maritimus*) age based on an epigenetic DNA methylation clock**

Susannah P. Woodruff^1^, Milda Milčiūtė^2^, Juozas Gordevičius^2^, Robert Brooke^2^, Todd C. Atwood^3^

^1^Marine Mammals Management, US Fish and Wildlife Service, Anchorage, AK 99503, USA.

^2^Epigenetic Development Clock Foundation, 1124 W Carson St, Torrance, CA 90502, USA.

^3^U.S. Geological Survey Alaska Science Center, Anchorage, AK 99508, USA.

***Supplementary material***

***Estimating age based on DNA methylation levels***

Table S1. Number of samples of known and tooth age bears by polar bear subpopulation, sex, and age (years). Age zero represents cubs-of-the-year that are less than 1-year old. Chukchi Sea = CS, Southern Beaufort Sea = SBS

|  | Known age | | | | Tooth age | | | |
| --- | --- | --- | --- | --- | --- | --- | --- | --- |
| Subpopulation | CS | SB | CS | SB | CS | SB | CS | SB |
| Age (years) | Male | Male | Female | Female | Male | Male | Female | Female |
| 0 | - | 4 | - | - | - | - | - | - |
| 1 | 3 | 2 | 4 | - | - | - | - | - |
| 2 | 3 | - | 3 | - | - | - | - | - |
| 3 | - | 3 | 1 | 3 | 1 | - | 1 | - |
| 4 | - | 4 | 2 | 3 | 2 | - | - | - |
| 5 | - | 3 | - | 4 | 2 | - | 2 | - |
| 6 | 1 | 1 | 2 | 2 | 1 | - | - | - |
| 7 | - | 3 | - | 3 | 3 | - | 3 | - |
| 8 | 1 | 3 | - | 4 | 3 | - | 2 | - |
| 9 | - | 1 | - | 4 | 2 | - | 1 | - |
| 10 | - | 5 | - | 4 | 2 | - | 1 | - |
| 11 | - | 4 | - | 6 | 3 | - | 4 | - |
| 12 | - | 1 | - | 3 | 3 | - | 2 | - |
| 13 | - | 3 | - | 4 | - | - | 2 | - |
| 14 | - | 2 | - | - | 1 | - | 2 | - |
| 15 | - | 1 | - | 1 | - | - | 2 | - |
| 16 | - | - | - | 1 | 2 | - | 2 | - |
| 17 | - | - | - | 1 | 4 | - | 2 | - |
| 18 | - | - | - | 1 | 1 | - | 2 | - |
| 19 | - | - | - | 1 | - | - | 2 | - |
| 20 | - | - | - | - | 1 | - | 1 | - |
| 21 | - | - | - | 1 | - | - | - | - |
| 22 | - | - | - | 2 | 1 | - | 1 | 1 |
| 23 | - | 1 | - | - | 2 | - | - | 1 |
| 24 | - | - | - | - | 2 | - | 1 | - |
| 25 | - | - | - | - | 1 | - | 1 | 1 |

Table S2. Specific cytosine-guanine (CpG) sites retained by the final elastic net regression model to estimate DNA methylation age from blood samples of polar bears (*n*  = 111) and the associated importance values.

Table S3. Principal component preprocessing estimates, including rotation and coefficients for each principal component.

Figure S1. A comparison between actual time between test samples (years) and the difference in estimated age via analysis of the level of DNA methylation (DNAm age) for polar bears captured and sampled in multiple years at known time points in the Chukchi (2008–2017; *n* = 34) and southern Beaufort (1987–2016; *n* = 3) Seas. Samples all came from bears with ages initially estimated via cementum annuli. The solid line represents the linear regression line fit to the data. The dashed line represents a 1:1 reference line where time between samples and difference in DNAm age are the same.

Figure S2. An examination of the correlation between the ages predicted by the polar bear specific clock and four universal clocks in the Chukchi Sea (CS) and southern Beaufort Sea (SBS) subpopulations.

.
